# Public Policies for Fluoride Use in Colombia and Brazil before and during the Adoption of the Right to Health

**DOI:** 10.3390/ijerph20032058

**Published:** 2023-01-23

**Authors:** Laura Jackeline García Rincón, Paulo Frazão

**Affiliations:** Department of Politics, Management and Health, School of Public Health, University of São Paulo, São Paulo 01246-904, Brazil

**Keywords:** fluorides, dental caries, public policy, dental public health, prevention

## Abstract

The use of fluorides is essential in the prevention of dental caries, considered to be the main dental public health problem. The formulation and implementation of public health policies can vary from country to country, depending on multiple factors. This study aims to analyze the interaction model between the knowledge produced about the use of fluorides and its implementation through public policies in two South American countries until the period of constitutional reform in each country. A narrative review was conducted with a systematic search of scientific articles and normative devices regarding the use of fluorides in public health in each country during the period prior to the implementation of the right to health in the Constitution. In both countries, there was an intense interaction among governmental organizations, researchers, academic and professional leaders, and companies involved in sanitation and salt production. Fluoride use strategies in Brazil and Colombia after an initial stage of similar characteristics began to differ in terms of public policy options for systemic fluoride use. In Brazil, the option was to adjust the concentration of fluoride in the water, while in Colombia, the addition of fluoride to table salt was consolidated as a public policy.

## 1. Introduction

Oral diseases are an important cause of morbidity and mortality, affecting about 3.5 billion people worldwide [1]. Among oral diseases, untreated dental caries is the main dental public health problem. Although preventable, it is the most common non-communicable chronic disease in the world, reaching an alarming prevalence of 90% in low-income countries [1,2]. When dental caries progresses, there is an irreversible loss of tissue, which can cause intense pain and even tooth loss, thus affecting the functionality, self-esteem, quality of life and well-being of those who suffer from it; moreover, its treatment is costly [2,3].

Among the public health strategies for dental caries control, fluoride has been widely investigated since the beginning of the 20th century, when its anti-caries effect in drinking water was recognized [4,5]. Since the mid-20th century, several methods of fluoride use in the population have been developed: collective ones, such as water, milk and salt; professionally administered ones, such as gels and varnishes; and those for self-use, such as dentifrices and mouthwashes. The most cost-effective and recommended strategies are those where population exposure to fluoride is regular and in low concentration to ensure its constant presence in the oral cavity; this decreases demineralization and activates the remineralization of the tooth enamel surface [6]. Its application can occur through specific programs funded by the State, and also through regulatory arrangements involving the market for fluoride products and dental services [7].

The formulation and implementation of these public health policies can vary from country to country. They depend on multiple factors, among which the constant interaction between national and international public health organizations and scientific knowledge, that is, the evidence or information available from reliable sources also of national or foreign origin [8,9].

That interaction between scientific production and public policies can be explained by different models that vary according to the premise on which they are founded: the knowledge-driven model is based on the notion from the natural sciences that if there is knowledge, it should be used; the problem-solving model derives from a specific problem that requires the study of a specific solution; the interactive model where knowledge is only one of the factors involved alongside accumulated experience, political vision, stakeholder pressure, social technologies and judgment; the political model in which knowledge is employed to support a political position; the tactical model in which knowledge is used to provoke a delay in order to avoid responsibility for undesirable outcomes; the enlightenment model that emphasizes the indirect influence of knowledge derived from concepts and theoretical perspectives of the human and social sciences on the policy-making process [10]. Investigating the model that guides the interaction between scientific knowledge and public health policies implementation is key to understand how evidence is used in practice and to provide a broader view of past choices and the current and future challenges linked to this interaction.

In the second half of the 20th century, Brazil and Colombia had insufficient availability of professionals to meet the high need for dental care of the population, and became a reference in the use of fluorides as a public health policy in Latin America. Before that, the use of fluorides in both countries was incipient and limited to topical administration methods in school programs. Both countries went through constitutional reform processes between the late 1980s and early 1990s, in which the right to health and the state’s responsibility in ensuring this right were inserted.

Part of a broader project aimed at producing a critical and comparative analysis on the trajectory of scientific production and policy guidelines regarding fluorides use as a public health strategy in two South American countries, this study aims to analyze the interaction model between the knowledge produced on the use of fluorides and its implementation through public policies in Brazil and Colombia until the period marked by the constitutional reform of each country. The hypothesis to be verified is that, while in Brazil an interactive model with strong participation of internal agents predominated, in Colombia external participation was decisive in the interactive model.

## 2. Methods

A narrative review was carried out with a systematic search of scientific articles and the normative devices regarding the use of fluorides in public health in each country during the period prior to the insertion of the right to health in the 1988 Federal Constitution in Brazil and in the 1991 Political Constitution of Colombia. This type of study allows the integration of information published in different sources, and the synthesis of a global view to propitiate the understanding of the problem. The selection of sources and the search strategies were guided by the following question: What are the relationships between the characteristics of the scientific production conveyed in the form of scientific articles and normative devices of national scope in each country?

In relation to the scientific literature, LILACS (Latin American and Caribbean Literature on Health Sciences), MEDLINE (Medical Literature Analysis and Retrieval System Online) via PubMed search, SciELO (Scientific Electronic Library Online), BVS (Virtual Health Library) and Scopus databases were selected. The searches for each country were conducted in February 2022 and were adapted to the available resources and characteristics of each database. The index terms (fluorine OR fluorides) AND (policy OR program) were used. This was conducted in order to cover fluoride both as a chemical element (fluorine) and as a chemical compound combined with other elements (fluoride), since in documents it can be found in both forms.

Inclusion criteria include scientific articles published in English, Spanish or Portuguese from 1960 to 1992 for Brazil and from 1960 to 1990 for Colombia about fluoride use in public health strategies, and normative devices or guidelines of official character and national scope. The time intervals were thus defined to cover the period prior to and the years surrounding the constitutional reform in which the right to health was included in the constitution of both countries. Although the period prior to the constitutional reforms is the main period of interest, the years around the promulgation of each constitution were included based on the notion that a constitutional reform, rather than being a one-off moment, expresses a period of transformation of a country marked by proposals and discussions in various spheres, which may result in outcomes of debates initiated in the previous period. Technical documents, theses, dissertations and course completion papers, documents with no author, records with repetitive information or that did not present results or information relevant to the research focus were considered as exclusion criteria. The search keywords are detailed in Table 1.

Regarding the normative devices, official sources of information and documentation of the institutions with competencies at the national level in the areas of health and surveillance with responsibility for conducting health policies and for health protection and regulation policies in each country were consulted. For Brazil, the advanced search for legislation on the official website of the federal government, the National Health Surveillance Agency (ANVISA), the National Health Foundation (FUNASA), and the repositories of the Information System of the National Congress (SICON), the SAÚDE LEGIS, and the Virtual Health Library of the Ministry of Health (BVSMS) were used. For Colombia, the official website of the National Institute for Drug and Food Surveillance (INVIMA) and the National Institute of Health (INS), and the repositories of the Unified System of Regulatory Information of the Colombian State (SUIN-Juriscol) and Digital Institutional Repository of the Ministry of Health and Social Protection (RID) were consulted.

The searches were conducted in December 2020 and were adapted to the available resources and characteristics of each data source. The terms fluoride, fluoridation, water quality, fluoride, dentifrice, rinse aid, salt for human consumption, oral health and derivatives were used. Table 2 details the strategies and the number of records obtained. The characteristics of the included publications were organized with a Microsoft Office Excel 2019 spreadsheet.

Based on titles and abstracts, records were selected for full reading. After the full-text reading by the two examiners, the scientific and normative documents for inclusion were selected. No records outside the subject and/or period of interest were included. One record obtained in the search for Colombia that referred to Brazil was transferred. All searches were performed by two researchers, results were discussed, and discrepancies were resolved by consensus.

### Data Analysis

The scientific articles were classified according to the vehicle of fluoride administration: water, salt and topical use, differentiating between rinses, toothpastes and applications of solutions and gels. The objectives and main aspects related to methods, results and final considerations were extracted.

The normative documents were read in their entirety and the data were extracted according to the following categories: title, type of document, date, responsible body, number of pages, subject, nature, topic (fluoride), main guideline or recommendation or statement, vehicle of administration, type of fluoride and observation.

The interpretation of the content was based on the different forms of interaction between scientific production and public policies, highlighting the interactive model, through which it is recognized that multiple parties play an active role and that there is a direct and indirect dialogue between policy design and scientific production. The policy-making process, from evidence to decision-making, is not linear but is configured in a series of not-always-aligned interconnections that challenge the course of policy [10,11].

## 3. Results

**Figure 1:**
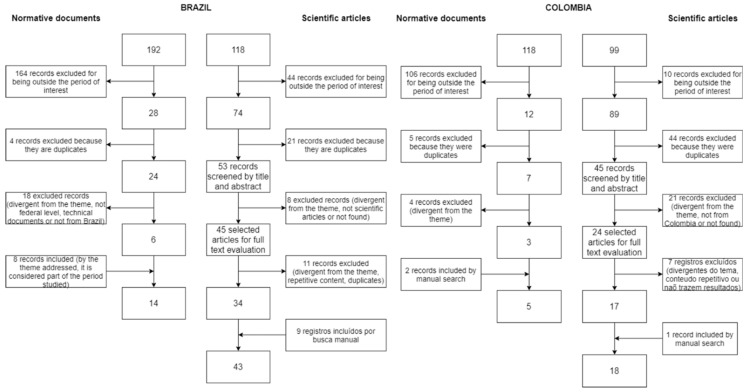
Flowchart of the records of scientific production and normative documents on the use of fluorides in Brazil and Colombia. Period of 1960–1991.

A total of 61 scientific articles and 19 normative documents were included. The material selection flowchart for each country is presented in Figure 1.

### 3.1. Colombia

The scientific articles found in the literature concerning Colombia are described in Table 3. In the period from 1966 to 1990, 18 publications were identified, of which 6 were literature reviews, 4 were food survey and nutritional status studies, 2 were studies on the prevalence of dental caries, and 3 were effectiveness studies, 2 of them evaluated the effect of a program to adjust the concentration of fluoride in water and the other on salt. The remaining three were about water fluoridation technology in a rural community, trace element concentration in drinking water and a study of fluoride sources for use in different vehicles.

The first initiative to use fluorides in strategies for the prevention and control of dental caries in Colombia dates back to 1948, in Bogota, with the topical application of sodium fluoride in the school population, which led to a 28% reduction in dental caries [26]. Five years later, under the direction of the Inter-American Cooperative Health Service (a special autonomous service created in 1942 by the Ministry of Health, and aligned with the interests of the United States), the first experience of water fluoridation began (Girardot, Cundinamarca, 1953), but the measure was suspended seven years later. Bogotá and Cali implemented water fluoridation in 1962, but a few years later, both initiatives also suffered interruptions. The longest successful experiences that best interacted with administrative, economic, technical and public opinion difficulties were in the cities of Manizales, from 1969 to 1986, and Medellín, from 1969 to 1989 [13,26,29]. These initiatives were driven by the National Aqueduct Fluoridation Program, launched in 1969 by the Colombian government, as a result of the epidemiological information produced by the first prevalence study of oral morbidities (Investigación Nacional de Morbilidad Oral 1965–1966) [26,30]. Instability in the availability of the fluoride substance, lack of interest by local authorities, and lack of equipment maintenance hindered the continuation and expansion of the program [26]. Despite the efforts of the Oral Health Division of the Ministry of Health, coverage benefited about 11% of the Colombian population in 1981 [25].

Between 1963 and 1972, a salt fluoridation study was developed in four communities in the department of Antioquia. An alternative water fluoridation method was sought that would be feasible and effective in preventing caries. It was argued that, in the face of the high prevalence of caries, salt could work because most of the population still lived in rural areas and did not have access to drinking water or an adequate supply system [12]. In the same vein, salt was a food with wide consumption and coverage and lower operating cost. The site chosen for the study in Colombia had high caries rates and low natural fluoride levels in water, and the inhabitants’ diet was high in foods derived from sugar cane. Furthermore, the production and distribution of salt in the country, being a government monopoly, were centralized, which would facilitate the application and management of the measure [16]. The study concluded that the effectiveness of fluoride salt was similar to that of fluoridated water as long as the concentration of fluoride in the salt was adjusted to achieve urine excretion levels similar to those observed in areas with optimal fluoridated water. The results showed preventive power in the order of 60 to 65% and viability to achieve a stable and homogeneous mixture of fluoride in cooking salt. Salt would be a feasible vehicle to provide the population with access to fluoride in adequate concentrations, especially those lacking an adequate public water supply. The method was simple, safe and low cost. The optimal dose of fluoride concentration in salt was 200 ppm F to prevent caries without the risk of fluorosis [12,16,23,24].

The initiative to adjust the fluoride content in the water in the city of Medellin was accompanied by a comprehensive prevention program that included topical self-application of 2% sodium fluoride solution (with the Bojanini technique) and educational activities. Because of the good results, the Medellin program was applied in public schools throughout Colombia under the name Dental Preventive Action, including four activities: oral health education, plaque control, teaching oral hygiene techniques and self-application of 2% sodium fluoride [25].

In 1982, fluoride mouthwashes were mentioned for the first time in the Colombian scientific literature. At the time, the aim was to reach the WHO goal for the year 2000 through disease prevention and primary health care. A study showed that the natural fluoride reserves in the country were high and sufficient to produce fluoride compounds and even to export them [28].

The normative documents regarding fluoride use for caries control in Colombia are described in Table 4. Five documents were highlighted between 1978 and 1984, all with involvement of the Ministry of Health. Two were concerned with salt fluoridation, one being Resolution 2772 of 1978, which created a committee to study the feasibility of salt fluoridation in Colombia. The committee was made up of representatives of the Ministry of Health, the National Planning Department, the University of Antioquia and Concesión Salinas. The other document is Decree 2024 of 1984, issued after the recommendations of the committee, which ordered the fluoridation of salt for human consumption in the national territory at a concentration from 180 to 220 ppm F, in accordance with the concentration found to be effective in the study in Antioquia, and complying with the requirements of Icontec Standard 1254 in force at the time. The normative does not specify the type of fluoride that can be used in the fluoridation process, although the results of the salt fluoridation study in Antioquia showed the effectiveness of two fluoride compounds [23]. Of the three documents concerning fluoride in water, one is concerned with water for agricultural use and the other two with water for human consumption. Article 73 of Law 9 of 1979, enacted by the Colombian Congress, made the Ministry of Health responsible for approving water fluoridation programs and operating criteria, as well as inspecting compliance with hygiene and safety standards in water treatment plants. Decree 2105 of 1983, which partially regulated Title II on water potabilization of Law 9 of 1979, in its article 17 specified that the fluoride concentration in the water should be controlled according to the average temperature of the location. Decree 1594 of 1984 cited the quality criteria that water should have for its different uses, determining as methods of analysis of fluoride in water the use of specific electrode, SPADNS and alizarin.

Since 1967, studies on salt and water fluoridation were observed. In the 1970s, the interactions between policy makers and researchers unfolded into regulations directed at salt fluoridation.

### 3.2. Brazil

The scientific articles related to Brazil are described in Table 5. From 1964 to 1991, 43 publications were identified, of which 21 were effectiveness studies, 7 were reviews, 5 were monitoring studies, 4 were studies on the use of fluoride in school programs and 3 on adverse effects of fluoride. The remaining three were about economic aspects of fluoridation, advocacy of fluoridation and effects of fluoridation cessation.

The first initiatives of fluoride use at a population level in Brazil occurred in the early 1950s. The school population of the city of Aimorés benefited from a topical application program of 2% sodium fluoride solution in 1952. One year later, the water supply system in Baixo Guandu, in the Espírito Santo state, had the fluoride concentration adjusted for caries prevention. Both programs were implemented by the Special Public Health Service (in Portuguese, Serviço Especial de Saúde Pública—SESP), a federal agency created in 1942 under an agreement with the US [36,41]. There was an important interaction between the professionals in sanitary engineering and sanitary dentistry. Studies on the effectiveness of water fluoridation between 0.7 and 0.9 mg F/L continued to show results similar to those obtained in the US and Canada [39,45,47,49,60,61,63,64,69,70,74,75]. In 1982, a study showed that the occurrence of dental caries was higher than expected in schoolchildren aged 7 to 12 years in the city of Araraquara, SP, sixteen years after the implementation of water fluoridation. Inconsistent data from the water supply company strengthened the hypothesis of discontinuity in fluoride concentration at an optimal level in water [55].

The topical use of fluorides in school children has been studied since the early 1980s. Research on the composition, stability and reactivity of and optimal fluoride concentration in toothpastes was published [51,67,68,73]. At the beginning of the following decade, the results of salt fluoridation research outside Brazil and the support of the Pan American Health Organization (PAHO) motivated the proposal of the Ministry of Health to fluoridate salt in the country. The elaboration of the Program, in a centralized way, was incompatible with the political context marked by a health reform that sought debate, participatory construction and decentralization of health actions and services. The measure was strongly opposed by the National Health Council and technical experts in the area, who encountered several practical barriers to the implementation of the strategy: the commercialization of salt would have to be differentiated according to regions because of the presence of fluoride in the water and the variation of the average daily salt consumption in the territory. It was argued that the priority for these regions at that time was to expand the water supply network and improve the ongoing water fluoridation, and that salt was harmful to the population’s health because of physicians’ fight against hypertension. Moreover, there were many doubts about how to prevent fluoridated salt from being sold in municipalities with optimal levels of fluoride in the water, and how to ensure an effective system of monitoring and correction of concentrations above or below the optimal level of the salt marketed in order to decrease the risk of fluorosis. The outcome was the revocation of the measure’s implementation and the reaffirmation of water fluoridation as the main public health strategy in the country [56,77].

The normative documents referring to the use of fluorides for caries control in Brazil are described in Table 6. During the period studied, fourteen documents were found between 1968 and 1992, of which five involved water fluoridation, including Law 6050 of 1974, approved by the National Congress, which made fluoridation mandatory wherever there was a water treatment plant, and Ordinance 635 of 1975, which established the norms and standards for its execution. Another six ordinances, whose responsible bodies were the Ministry of Health and the National Institute of Food and Nutrition, dealt with the issue of standards for the fluoridation of table salt and its revocation. Regarding topical fluorides, two regulations were approved by the National Health Surveillance Secretariat of the Ministry of Health, establishing quality criteria for mouthwashes and toothpastes. These standards were established in the late 1980s in a context where fluoride toothpastes were sold on a large scale.

## 4. Discussion

An overview of the relationship between the scientific production regarding fluorides use and the public policies derived from this knowledge in two countries considered reference in the use of dental caries control strategies in Latin America was presented. The examination of the material showed that, in both countries, there was intense interaction between governmental organizations, researchers, academic and professional leaders, and also between companies linked to sanitation and table salt production. Both countries were marked by a legacy of international cooperation activities with the US in the areas of health and sanitation that had intensified between the 1940s and 1960s. In the 1970s, both had insufficient dental resources and high caries experience measured by the DMFT at ages of 12: 7.1 in Colombia, and 8.6 in Brazil [93,94,95]. Similarities were observed in the scientific production, such as interest in studies of the effectiveness of interventions, cost analysis and availability of fluoridating substances, and also differences, highlighting in Colombia interest in nutritional aspects and in Brazil interest in monitoring fluoride in dentifrices. The distribution of Brazilian studies was more diversified, with a greater number of researchers from different universities, when compared to Colombia. An additional important difference between the countries was that the salt fluoridation proposal supported by the PAHO found, in Colombia, a favorable ground for its implementation, while in Brazil, the interactions expressed themselves in disputes regarding the most appropriate public policy whose result, at the same time, represented a brake to the proposal of table salt fluoridation, served to reaffirm water fluoridation as a public policy option for systemic use. With this, it can be accepted the hypothesis that, while in Brazil an interactive model with a strong participation of regional and local internal agents predominated, in Colombia, external participation was decisive in the interactive model that proved to be compatible to describe the relationship between scientific production related to the use of fluorides and the public policies derived from this knowledge both in Brazil and Colombia.

The international context of the time regarding research was marked by the search for determining the optimal level of fluoride intake to obtain maximum protection against caries with a minimum risk of dental fluorosis and by studies of the effectiveness of topical methods [96]. As a strategy during World War II, the US government and 21 Latin American countries signed an agreement for political and cultural rapprochement and cooperation in health and sanitation within the framework of good neighbor geopolitics [97,98]. In Brazil, the Special Public Health Service (SESP) was created, while in Colombia, the Inter-American Cooperative Health Service (SCISP) was structured to mediate relations between the US Institute of Inter-American Affairs (IIAA) and the Colombian Ministry of Labor, Hygiene and Social Welfare. It was a separate but dependent entity of the Ministry that would help in matters of cooperation aimed at obtaining raw materials for the war and also in the defense of hemispheric security [99,100]. Both institutions in Brazil and Colombia promoted the creation of water supply systems and made efforts towards water fluoridation in the region.

In Latin America, the Pan American Sanitary Bureau (PASB), headquartered in Washington, led the health actions in the region. The concern for the implementation of water fluoridation in Latin America was discussed internationally. Since 1956, the PASB collaborated by advising countries with problems in the process of fluoridation of public water supply because of the high costs of importing fluoride compounds from the US and Europe, and the need to explore natural sources and reserves of fluoride in the countries [101]. Starting in 1963, the PASB began to study salt fluoridation as an alternative method to water fluoridation, due to the economic impediments that some regions had to implement the measure, and selected Colombia for this purpose.

The history of both countries after World War II was marked by the incorporation of incentives aimed at industrialization in the context of agro-exporting economies. Starting in the 1970s, a process of democratic recovery and proposals for constitutional reform began to take shape in opposition to the context of human rights abuses, violence and profound social inequalities that characterized the countries. This aspiration for greater social participation was expressed in the constitutions of 1988 in Brazil and 1991 in Colombia, which emerged in the so-called new Latin American constitutionalism (NLC), characterized by the expansion of rights, readjustment of the functions of the judiciary and increased popular participation. Brazil was experiencing the end of dictatorship and emerging social movements were demanding a Constituent Assembly favorable to the redemocratization of institutions. The result was the approval of a Constitution that expanded the powers and granted political, administrative and financial autonomy to the Federal District, the States and the municipalities. This contributed to the active manifestation of administrators and professionals in the public dental service against the imposition of salt fluoridation in Brazil in the North and Northeast regions, where the program would initially be implemented. In the Colombian case, there were high levels of violence resulting from internal armed conflict and drug trafficking, in addition to growing urbanization and unemployment. In 1990, an inclusive Constituent Assembly was convened, with previously marginalized sectors participating in the construction of a Constitution that provided opportunities for equity and democratic participation, and consolidated the decentralization and autonomy of the territorial entities [102].

The comparison of the cases presented in this paper confirms the external influence exerted by international agencies subject to their own challenges and interests whose role can be more or less relevant depending on the context of internal interactions in each country and corroborates the notion that cooperation activities between countries can occur in a context of asymmetric relations of interdependence [103]. In Brazil, the external influence found a context of interactions marked by a health reform that involved greater social participation in the design of health policies [104], while in Colombia, we observed a favorable scenario for the interests of international agencies at the time due to the centralized production and distribution of table salt in the country.

Among the limitations of this study, the quality of the data sources can be considered. The documentary research involved the search for normative devices related to the second half of the 20th century. The cataloging and archiving of such devices depends on the documentation offices of public health institutions, and documents related to the topic might not have been retrieved due to the lack of indexing in the selected data collections. However, to avoid a possible selection bias, collections from different institutions and organizations in each country were investigated. Given the focus of the study, only national regulations were included, ruling out sub-national regulations in each country. However, the view taken seeks to show an overview of the country as a whole and not regional or local aspects that were not reflected at the national level. Furthermore, the study involved two South American countries, which limits the extrapolation of the results to other countries with different historical, economic and political conditions. Despite the limitations, the scientific information produced may help in the understanding of historical, political–institutional and health aspects linked to the use of fluoride in public health, including missed and seized opportunities aimed at protecting the oral health of the population in each country. As seen, public policies are not fixed; they vary and adapt according to time and context, and this type of analysis can contribute to assess the directions and indicate limitations and challenges regarding fluoride use policy in public health. Moreover, by bringing together a comparative analysis of two reference cases, the study can support the examination of the policy in other countries, both in Latin America and in other regions of the world. For other Latin American countries, the most recommended approach is to evaluate their own social, environmental, climatic, social, economic and political conditions and characteristics, in addition to measuring the natural fluoride content in the water, knowing the national sources of fluoride or the need for imports, as well as the caries prevalence levels in order to apply a strategy that meets the needs of the population. Future research may benefit from this study, using the information as a starting point for analyzing the course of fluoride use policy in public health.

## 5. Conclusions

In view of the analysis of the scientific literature and regulatory devices in both countries in the second half of the 20th century, it can be concluded that fluoride use strategies in Brazil and Colombia, after an initial stage of similar characteristics and based on fluoridation of public water supplies and topical application of sodium fluoride solution on the teeth of schoolchildren, started to differ in terms of public policy options for systemic use of fluorides. In Brazil, the policy adopted was to adjust the concentration of fluoride in public water supplies, while in Colombia, the public policy consolidated was the addition of fluoride to table salt or sodium chloride (NaCl). In both countries, the accumulated experience and pressure from stakeholders were as important as the scientific knowledge produced; however, while in the former an interactive model with strong participation of internal agents predominated, in Colombia, external participation was crucial for this interaction.

## Figures and Tables

**Table 1:** Search keywords in databases for Brazil and Colombia.

	Brazil		Colombia	
Database	Search Keywords	Records	Search Keywords	Records
BVS	flúor AND Brasil	56	flúor AND Colombia	38
LILACS	flúor OR fluoretos [Palavras] and políticas OR programas [Palavras] and Brasil [Palavras]	46	flúor OR fluoruros [Palavras] and políticas OR programas [Palavras] and Colombia [Palavras]	11
PubMed	(fluor*) AND (dental health) AND (Brazil)	10	(fluorine OR fluorides) AND (Colombia)	27
SciELO	fluor* AND Brasil	5	fluor* AND Colombia	0
Scopus	(TITLE-ABS-KEY (fluorine OR fluorides) AND TITLE-ABS-KEY (policy OR program) AND TITLE-ABS-KEY (brazil)) AND PUBYEAR > 1959 AND PUBYEAR < 1989	1	(TITLE-ABS-KEY (fluorine OR fluorides) AND TITLE-ABS-KEY (colombia)) AND PUBYEAR > 1959 AND PUBYEAR < 1992	23
Total		118		99

**Table 2:** Search strategies for normative documents related to the use of fluoride in public health and number of records obtained for Brazil and Colombia.

Brazil			Colombia		
Information Source	Search Strategies	Records	Information Source	Search Strategies	Records
Planalto	Advanced search with terms: flúor, fluoreto	35	Ministry of Health and Social Protection	Normativa (Manual search in ascending chronological order)	7
SAÚDE LEGIS	Topic: Flúor	4	SUIN JURISCOL	“sal para consumo humano” + Filter: Salud y protección social	2
SAÚDE LEGIS	Topic: Fluoretação	8	INVIMA	Normatividad/Índice por temas/Cosméticos (Manual search in ascending chronological order)	32
SAÚDE LEGIS	Topic: Qualidade da água	18	INVIMA	Normatividad/Índice por temas/Sal	2
SAÚDE LEGIS	Topic: Fluor	15	INVIMA	Normatividad/Búsqueda avanzada/texto: dentríficos	12
SICON	Fluoretação Filters: Gestão de Normas Jurídicas (Legislação Federal); Repositório de Documentos Legislativos	2	INVIMA	Normatividad/Búsqueda avanzada/texto: dentífricos	6
BVSMS	Legislação da Saúde/Legislação Básica do SUS	9	INVIMA	Normatividad/Búsqueda avanzada/texto: “enjuagues bucales”	10
BVSMS	fluoretos (Filter: Base de dados: Ministério da saúde)	4	INVIMA	Normatividad/Índice por temas/Agua	14
BVSMS	fluoretação (Filter: Data base: Ministry of Health)	9	INS	IQEN (Manual search in chronological order)	4
BVSMS	(flúor) AND (dentifrício) OR (enxaguatório) OR (verniz) + Filter: portuguese. Data base: Ministry of Health	11	INS	Technical-scientific publications/protocols and notification sheets/Event: Fluoride exposition	6
ANVISA	Portal ANVISA/Legislation/dental hygiene; tipo de atos: Todos; Topics: Cosméticos	45	INS	Manual search	3
ANVISA	Thematic Libraries: *Alimentos (água envasada)	4	RID	Searches with key-words (flúor, fluoruro, sal)	20
ANVISA	Thematic Libraries: *Cosméticos (dentifrícios e enxaguatórios bucais)	26			
FUNASA	Portarias FUNASA (manual search in ascending chronological order)	2			
Total		192	Total		118

**Table 3:** Scientific production related to the use of fluorides in the prevention of dental caries in Colombia in the period prior to the constitutional reform.

Author–Year	Study Design/Vehicle	Aim	Methods/Development	Results/Final Considerations
Restrepo, D. (1967) [12]	Review/Salt	To show salt fluoridation as an alternative to water fluoridation.	The pioneering study in Colombia is described with its objectives, methods and preliminary results.	Fluoridated salt is presented as a viable method for communities and regions where the water system does not allow for the implementation of fluoridation processes due to the high cost or lack thereof. Fluoridated salt can reach rural areas easily and at a minimal cost; the processes are simple, safe and low-cost. A uniform mixture of salt and fluoride was achieved, the average daily salt consumption was established to calculate the fluoride dose and annual studies will show the effectiveness of the method against caries.
Rozo, G.J. (1969) [13]	Effectiveness study/Water	To show the evaluation of a program to add sodium fluoride to the water supply system of a Bogota neighborhood.	The background and circumstances that have caused the city to suspend water fluoridation are analyzed. The DMF of Bogota in 1962 (before water fluoridation) and of Santa Isabel (Bogota district) in 1968 (6 years after water fluoridation) were compared with the data of Cachipay (rural area without fluoride in the water) in 1968.	There was a 45% decrease in dental caries after 6 years of continuous application of sodium fluoride in water.
Mejía et al. (1969) [14]	Dental caries prevalence study/Water	To describe the results of a study in which the low prevalence of caries in a community is not attributable to fluoride, since it was not added to the water and the content of it in natural water was negligible.	In 1966, an epidemiological caries survey was conducted in Heliconia, which included all school children aged 8–14 years (n = 267). The DMFT and dmf indices were used.	The very low levels of caries and the good condition of the teeth even without dental care are remarkable. There are more children with DMFT equal to zero than in similar communities. In the county, there is possibly no racial, genetic or nutritional factor that influences the low prevalence and incidence of caries. There could be an extrinsic factor present in the water acting at a systemic level. It is possible that there is something other than fluoride ions that has the power to prevent caries.
Vélez et al. (1970) [15]	Fluoridation technology/Water	To describe the experience with a highly simplified and cost-effective fluoridator, transferable in its present form, or with very feasible modifications, to other rural areas that, from not having this means, would be deprived of fluoridation measures for small water supply systems.	Due to the lack of electrical energy in the municipality of San Pedro, a simple and economical gravity fluoridation system was chosen. The procedure for the installation and operation of the NaF dosing equipment designed by the University of Antioquia are explained, adopting 1 ppm F according to the average annual temperature.	The cost for a community of 4500 inhabitants would be COP 1.33 (USD 0.07) per person per year. The fluoridator described worked with proven accuracy during the four years of the trial. The cost of the accessories and tanks is COP 2420 (USD 138.28). The components of the fluoridator are simple, easy to acquire, economical and safe. This makes it feasible to build and use in any environment. In addition, operation can be performed by an ordinary worker, with easy-to-understand instructions and no risks.
Restrepo et al. (1972) [16]	Review/Water, Salt	To investigate the feasibility and effectiveness of adding fluoride to table salt as an alternative caries prevention measure.	In 1963, a census was taken in the four selected communities, in addition to radiographs, clinical and laboratory examinations; dietary survey, determination of average daily salt intake; and caries survey and DMF index. The method for mixing salt and fluoride was studied, considering the optimal concentration of 1 ppm.	The feasibility of adding calcium fluoride or sodium fluoride to salt in a stable mixture was demonstrated. Preliminary results indicate a reduction in caries only in communities receiving fluoridated water and salt. In the future, a study will be conducted on the economic feasibility of the measure for large geographical areas.
Rothman et al. (1972) [17]	Dental caries prevalence study/Water	To determine whether children in the municipality of Heliconia really have an unusually low caries prevalence, and if so, to identify the causes.	All schoolchildren between 12 and 17 years old were examined (n = 302; 148 from Heliconia and 154 from Don Matías). Teeth were recorded as erupted or unerupted, permanent or deciduous, present or lost.	Children in Heliconia have notably less caries than those in Don Matías. Both municipalities have similar but lower than optimal fluoride concentration in drinking water. No known factors influencing caries activity could explain the low caries prevalence in Heliconia, although the role of undetermined or undiscovered environmental factors could be important. Factors such as fluoride, oral hygiene, diet, genetics or location do not seem to be the cause of the marked difference in caries prevalence.
Vélez et al. (1973) [18]	Nutritional status study/Salt	To correlate the values found for height, weight and bone growth in communities with different socioeconomic levels and with different nutritional intakes.	In the four communities, weight and height were measured in two groups: the first formed by the totality of the schoolchildren (n = 2475) and the second by the population from 0 to 18 years from the control families. Hand and wrist radiographs were taken in the latter group (n = 1513). For control purposes, 366 measurements were taken in schoolchildren from a private school in Medellin.	There is growth and development deficiency in schoolchildren from the four communities in the salt fluoridation study when compared to children from a private school with a high socioeconomic status. Studies on the diet of these communities suggest that the protein and calorie intake is responsible for growth retardation and malnutrition.
Glass et al. (1973) [19]	Trace element concentration/Water	To evaluate trace element concentrations in drinking water samples from Don Matías and Heliconia municipalities, and relate the observed differences to the marked difference in the prevalence of dental caries.	A total of 41 samples were taken in the Heliconia houses and 50 in Don Matías. Trace element analyses were conducted by emission spectroscopy. Soluble fluoride levels were also analyzed using an electrode.	A total of 13 trace elements were studied. The concentrations of calcium, magnesium, molybdenum and vanadium were higher in the Heliconia samples, while the concentrations of copper, iron and manganese were higher in Don Matías. None of the samples contained more than 0.3 ppm F. The average fluoride level was less than 0.1 ppm in each municipality.
Hernández et al. (1973) [20]	Food survey/Salt	To show the results of the food survey conducted in the two communities receiving fluoride salt within the salt fluoridation study (Armenia and Montebello) and correlate these data with the incidence of dental caries.	The survey was conducted in 7 days with the method of the Institute of Nutrition of Central America and Panama (INCAP). The average daily nutrient intake per person and the nutritive value of the average diet by social class and by community were calculated.	There are deficits of the various nutrients in the population evaluated. Most belong to the poor and very poor socioeconomic classes, where nutritional intake is much lower. The consumption of dairy products, eggs, meat and vegetables with high-biological-value proteins is minimal. This deficiency is reflected in the growth and development disorders of individuals. The diet of the communities is based on cereals, sugars, tubers, plantains and a minimal amount of fat.
Hernández et al. (1974) [21]	Food survey/Water	To show the results of the food survey conducted in the municipality that receives fluoridated water (San Pedro) and the control, which does not receive fluoride (Don Matías). Then, a comparison, from a nutritional point of view, of communities that receive fluoride salt and establish the possible role of diet in reducing dental caries.	Considering gender and age of the sample, as well as socioeconomic level and family income, the consumption of different food groups and their proportion in the average diet were observed. These data were contrasted with the nutritional recommendations for nutrient intake.	Both communities were found to have a low intake of high-biological-value proteins, a more or less adequate intake of calories and a very low intake of fats, calcium and various vitamins. Most people were poor or very poor, whose nutritional intake is extremely below the recommended levels. The same was observed in Armenia and Montebello.
Hernández et al. (1974) [22]	Food survey/Salt	To know the daily salt intake, per person and per day, in the four communities within the study, in order to add fluoride to table salt in a dose capable of preventing caries in a manner equivalent to the amount used in drinking water.	The study was conducted by social class due to the variation in consumption according to economic capacity. The weight of the food was measured during 7 days paying special attention to the salt consumed by the family at the beginning and at the end of the evaluation.	It is necessary to add 1 mg F for every 10 g of table salt to prevent caries. The salt consumption in the 231 families studied ranged from 3 to 30 g. The average salt intake was similar in the four communities.
Mejía et al. (1976) [23]	Effectiveness study/Salt	1. To study the effectiveness of common salt as a carrier of fluoride to prevent dental caries. 2. To compare the relative effectiveness of sodium and calcium fluorides as salt additives in preventing caries. 3. To establish the optimal dose of fluoride in table salt to achieve the maximum level of preventive action without risk of fluorosis. 4. To compare the efficacy of fluorides administered through table salt with those applied in water.	Four communities similar in their socioeconomic conditions, nutritional and health status, geographical location and minimal migration movement were selected. Since 1965, fluoride salt has been administered in two communities: Armenia (calcium fluoride) and Montebello (sodium fluoride); in San Pedro, fluoridated water was distributed and Don Matías was the control community that was left without receiving fluoride. Epidemiological surveys of dental caries were conducted annually from 1964 to 1972, to observe the variations presented.	The results confirm the discovery of a caries prevention method: adding fluoride to table salt. This is a viable method, since it is a food staple, low cost and easy to obtain. Fluoridated salt can prevent caries by 60 to 65%, similar to fluoridated water. A dose of 200 mg F/kg of salt is efficient.
Marthaler et al. (1978) [24]	Review/Salt	To expose some studies on salt fluoridation and show the limitations of this method.	Studies from Switzerland, Colombia, Spain and Hungary on salt fluoridation are reported. Difficulties are pointed out, such as: the method of producing fluoridated salt is different in each country; the determination of optimal fluoride levels in salt may vary significantly according to the different means of access to the product and waste in food preparation. It is important that the excretion of fluoride in urine is 1 ppm F.	Fluoride ingested with salt prevents caries. The cariostatic effectiveness of salt appears to be equal to that of water when the fluoride concentration is adjusted to the levels excreted in urine. It is possible to make a stable and homogeneous mixture of salt and fluoride.
Herazo-Acuña, B. (1982) [25]	Review/Topical application, self-applications, mouthwashes, salt and water	To promote primary oral health care in as much as possible.	Current status of oral health in Colombia, considerations about dentistry, primary health care activities, water fluoridation and salt fluoridation (if the program were to be implemented, water fluoridation would have to be suppressed as one replaces the other).	Primary health care must be prioritized. The fluoridation of water or salt must be promoted in order to reduce caries by 50 to 60%. The combination of this with other measures will allow Colombia to reach the year 2000 with a 90% reduction in oral pathologies.
Herazo-Acuña, B. and Salazar-Oliveros, L. (1983) [26]	Review/Water, salt and topical applications	To show some aspects of preventive programs in Colombia and to highlight the country’s experience in applying preventive measures to avoid oral pathologies.	General history of fluoride use; experience with fluoride use in several cities in the country; salt fluoridation; National Program for fluoridation of water supply systems; preventive dental action (annual topical applications); projects to fluoridate salt in Colombia.	The article looks forward to the results of the research conducted at the moment, in order to establish a national salt fluoridation program in Colombia in the future.
Herazo-Acuña, B. (1984) [27]	Review/Topical applications, mouthwashes, water and salt	To condense as much information as possible on fluoride and make it a comprehensive reference material for dentists.	Natural state, procurement, compounds, toxicology of fluoride, clinical symptoms of acute fluoride poisoning, benefits of fluoride, mechanisms of action of fluoride on dental caries and the phases of incorporation of fluoride into the tooth.	In most countries of the world, numerous preventive programs against caries and periodontal diseases have already been carried out, based on fluoride intake and to date no pathology produced or derived from this process has been reported. The known cases of fluorosis are due to the ignorance, in some cities, of the fluoride content in their public supply water and timely defluoridation programs are not carried out.
Herazo-Acuña, B. and Salazar-Oliveros, L. (1985) [28]	Study of fluoride sources for use in different vehicles	Communicate to professionals, industry and interested sectors about the great opportunities Colombia has to become a fluoride producer and exporter.	Information from the Colombian Mining Inventory on fluorite (insufficient) and phosphate rock (the largest) is presented.	This study demonstrates the possibility of producing the full range of fluorides in Colombia, being technically feasible and cost-effective. The fluoride plant will produce social, economic and political benefits for a region of Colombia and for the country as a whole, helping to produce jobs.
Calle et al. (1990) [29]	Effectiveness study/Water, salt and topical applications	1. To determine the oral health status of the population under 20 years of age, after 20 years of the policy. 2. To determine if the results influence the coverage of oral health services. 3. To observe the behavior of caries and periodontal disease in order to reorient or modify technical–administrative norms. 4. Compare these results with future results with fluoride in salt.	In 1968, given the precarious oral health in Medellín, an alternative was sought to prevent caries on a collective level. Since 1969, an organized policy was based on prevention programs (water fluoridation, prevention activities and sealants) and assistance. This study coincided with the extinction of water fluoridation and the introduction of salt fluoridation.	It has been possible to control caries and periodontal disease, reducing the DMFT, dento-maxillo-facial anomalies and dental mortality, as well as the need for treatment and increasing the coverage of dental services. The number of people without caries increased. Early caries and periodontal disease control was achieved, meeting and exceeding the WHO and World Dental Federation (FDI) goals for the year 2000, so bolder goals were set for Medellin in the future.

**Table 4:** Normative documents related to the use of fluorides for dental caries prevention in Colombia in the period prior to the constitutional reform.

Title	Date	Institution	Vehicle	Topic	Orientation/Recommendation/Declaration
Resolution 2772 of March 27, 1978 [31]	1978	Ministry of Health	Salt	Creates the Committee for feasibility studies for the fluoridation of salt in Colombia.	Conduct studies and research necessary to decide whether to fluoridate salt in Colombia.
Law 9 of January 24, 1979 [32]	1979	Colombian Congress/Ministry of Health	Water	It dictates sanitary measures.	Art. 73. The Ministry of Health is responsible for the approval of the programs of fluoridation of water for human consumption, as well as of the compounds used to carry it out, its transportation, handling, storage and application of the methods for the disposal of residues.
Decree 2105 of July 26, 1983 [33]	1983	Ministry of Health	Water	Partially regulates Title II of Law 9 of 1979 regarding water potabilization.	Art 17. The content of fluoride as fluoride ion, F- should be controlled according to the average temperature of the environment.
Decree 1594 of June 26, 1984 [34]	1984	Ministry of Agriculture/Ministry of Health/National Planning Department	Water	Partially regulates Title I of Law 9 of 1979 regarding water uses.	Admissible quality criteria for the agricultural use of the resource: fluoride in quantities of 1 mg/L. The specific electrode, SPADNS and alizarin methods of analysis for fluoride are considered officially accepted.
Decree 2024 of August 21, 1984 [35]	1984	Ministry of Health/ Ministry of Economic Development	Salt	Determines the standards on iodization and fluoridation of salt for human consumption and regulates the control of its repackaging.	The fluoride content in salt must be in the proportion from 180 to 220 ppm. Salt packaging and containers must comply, among others, with the labeling requirements: fluoride and iodine content expressed in ppm.

**Table 5:** Scientific production related to the use of fluorides in the prevention of dental caries in Brazil in the pre-constitutional reform period.

Author–Year	Study Design/Vehicle	Aim	Methods/Development	Results/Final Considerations
Freire, P.S. (1964) [36]	Fluoride use in school programs/Water, topical application of sodium fluoride solution	Describe the incremental dental care program applied, combining curative and preventive services, which was approved as the standard for other communities in Brazil.	In Aimorés MG, two prevention methods were used: fluoridated water and topical application of 2% sodium fluoride. The plan had two phases: the initial program and the maintenance program.	After 5 years of operation, there were no accumulated dental needs and a significant increase in caries-free teeth.
Mello, C.F. (1966) [37]	Review/Water, topical application of sodium fluoride solution	Study the caries preventive and control measures that can be put into practice by dentists in their daily routine, in health care services and in private practices in Brazil.	It brings together much of what was known about caries and its prevention up to that point. The topics covered are caries basics, diet, microorganisms, fluoride, education and caries control. Regarding fluoride, the presence of fluoride in human and animal teeth and bones, stained teeth and caries prevention are described.	Water fluoridation can only be applied in communities that have a water treatment system and whose authorities are willing to support the measure; there are many communities that do not have such conditions. There is an urgent need to tackle the problem of caries with other preventive methods. Preventive measures should be put in place with the help of federal or local programs, such as health education, hygiene, fluoride tablets or topical applications.
Viegas, Y. (1970) [38]	Effectiveness study/Topical application of acidulated phosphate fluoride (APF) solution	Assess whether APF solutions, which have a proven effect on children, also have an effect on young adults, who already have mature enamel.	The study comprised a topical application of APF solution, containing 1.23% fluoride, to 75 undergraduate students with an average age of 20 years, themselves serving as controls.	There was a 27.66% reduction in the incidence of caries. The results allow the application of APF solution to young adults to be recommended. It would be desirable that this study be repeated in order to confirm the results obtained.
Viegas, Y. and Viegas, A.R. (1974) [39]	Effectiveness study/Water	Present and analyze the data on dental caries prevalence verified in the study of public water supply fluoridation in Campinas. To verify the dental caries reductions found in permanent and primary teeth during the ten-year period of water fluoridation.	Children from 4 to 14 years old were included in all four surveys. In the last three surveys, only children who have always lived in Campinas were examined. The indices used were DMFT, dmf and that of permanent first molars.	The caries prevalence reductions observed were 66% for permanent teeth and 53% for primary teeth. In children aged 6 to 10 years, 25% have no decayed primary teeth and 36% are in the same condition for permanent teeth.
Horowitz et al. (1974) [40]	Fluoride use in school programs/Topical self-application of APF solution and gel	Evaluate the effect of self-applications of APF in solution and gel, through supervised brushing.	A total of 566 children between 14 and 17 years old in São Paulo participated. Five study groups were formed: A (control) brushed their teeth with a fluoride-free prophylactic paste and then with a placebo solution; B brushed their teeth with the paste and then with APF solution (0.6%); C brushed their teeth with the same APF solution without brushing first with the paste; D brushed their teeth with the paste and then with APF gel (1.23%); and E brushed with the same APF gel without brushing first with the paste. Fifteen brushings were supervised at the school over 3 years.	There are a number of important non-controllable variables in the program: the actual conduct of the brushing sessions, exposure of the study population to fluoride (although some fluoride toothpastes are sold in Brazil, none of the compositions are similar to those accepted by the ADA or tested for efficacy), and the degree of oral care. After the 3 years of the study, there were incremental reductions in DMFS of 26, 26, 33 and 19% in groups B, C, D and E, respectively, compared to the control. Equal or even greater benefits were seen when fluoride was administered weekly or biweekly via mouthwashes. Whether the benefits are from supervised brushing or educational action remains to be seen.
Grinplastch, B.S. (1974) [41]	Review/Water	Recommend water fluoridation, which is one of the great advances in modern public health. Focuses on the compound fluorite or calcium fluoride.	Mentions the abundance of fluorite in Brazil, but the difficulty in using it because it is not water soluble. Reports the attempts and studies conducted to enable the use of fluorite in water fluoridation.	The incidence of caries can be reduced by about 65% by using water with fluoride content around 1 mg/L. Water fluoridation does not change any of the properties of water and is recommended by international health authorities as the only effective measure to prevent dental caries. The installation of fluoride compound factories is suggested.
Ando et al. (1975) [42]	Adverse effects of fluoride/Water	Describe dental fluorosis to estimate its severity in schoolchildren aged 6 to 14 years and make some considerations about the various forms of dental condition.	There were 175 schoolchildren, aged 6 to 14 years, all residents of a restricted area of Cosmópolis, SP. The water in this area comes from a semi-artesian well, opened in 1962, and contains about 9.5 to 11 ppm F. The average maximum temperature is 35.5°. The Dean fluorosis index was employed.	All degrees of fluorosis were present, but the moderate form was the most prevalent; there was no marked difference between the mean dental fluorosis rates in schoolchildren who ingested water soon after birth and those who started years later.
Saliba et al. (1975) [43]	Monitoring/Water	Check the differences that may exist in the determination of the concentration values of the fluoride ions existing in public supply waters; if differences exist, check if they have any positive or negative trend; and the importance of this trend.	A total of 32 water samples from public water supply systems with treatment plants or served by wells were evaluated. Given the existence of interferents that can induce errors in the determination of fluoride, a preliminary determination was made, then distillation of the samples and the final determination by 3 processes: Colorimetric methods SPADNS (Hach DR-El-AC) and Scott–Sanchis (HelligeAquatester and Nessler tubes).	The samples were within the recommended limits (0.6 to 1.5 ppm F). The fluoride ion determinations by the two methods differ from each other with a positive trend, since the Hach DR-EL-AC device shows higher results most of the time. For the most unfavorable case, which is that the difference of 0.11 mg/L always occurs, this does not cause any problem for the population’s health, and the additional costs are small, allowing both medium and small cities to control the fluoride concentrations in their water supply systems without major problems.
Ando T. (1975) [44]	Effectiveness study/Water	1. Study the caries prevalence in permanent teeth in schoolchildren residing in two regions with high and low fluoride content in the water. 2. To verify the possible difference regarding sex in caries prevalence in schoolchildren in the two areas. 3. Try to establish a relationship between the role played by fluoride, under fluorosis condition in reducing the caries prevalence in schoolchildren living in the area whose water supply has a high fluoride content.	We examined 324 schoolchildren from 7 to 14 years of age of both sexes. The sample was divided into 2 groups. The fluorosis group (n = 164) from the area with a high fluoride content (9.5 to 11 ppm) and the control group (n = 160) from the urban area, where the water supply contains about 0.05 ppm F. The mean DMFS index and clinical and radiographic examinations were used.	The caries prevalence observed in the fluorosis group was lower when compared to the control group. In both groups, there was no statistically significant difference between sexes in caries prevalence. The mean DMFS increased over time in both sexes and in both groups. Under dental fluorosis conditions, teeth were protected against caries both in those that calcified under this condition and in those that were influenced by an excess of fluoride after mineralization.
Freire, A.S. (1976) [45]	Effectiveness study/Water	Present the first results obtained with water fluoridation in Cachoeiro de Itapemirim, ES, after 6 years of implementation of the measure.	After an economic feasibility study, the Autonomous Service of Water and Sewage (SAAE) acquired the equipment to implement water fluoridation. The daily control is conducted by the Scott–Sanchis method. To date, fluoridation has not been interrupted and presents an average daily content of 0.8 ppm F.	In 1975, six years after the installation of water fluoridation in 1969, a notable reduction in caries rates is observed, despite not having alarming rates before fluoridation, due to the fact that there was reasonable school dental care.
Saliba, N.A. and Saliba, O. (1977) [46]	Effectiveness study/Topical application of APF solution	Compare methodologies employed by two researchers who obtained contradictory results on topical fluoride application.	The study started with 142 schoolchildren aged 7 to 10 years, of both sexes, and ended with 92. An acidulated fluoride solution (1.23% fluoride in orthophosphoric acid) was used after general prophylaxis.	The analysis of the results obtained revealed that topical application reduced caries incidence by approximately 16% with both one and two topical applications.
Rocca et al. (1979) [47]	Effectiveness study/Water	Compare the data collected on caries prevalence in Araraquara and in Guariba, SP, where the water is fluoride-free.	The sample was 860 schoolchildren from 7 to 12 years of age, of both sexes. The DMFT and DMFS indices were used.	Schoolchildren from Araraquara, where the water is fluoridated, had lower rates of DMFT, DMFS and loss of first permanent molars than those from Guariba.
Alcaide and Veronezi. (1979) [48]	Adverse effects of fluoride/Water	To investigate the presence of dental fluorosis in the city of Icém, measuring its extent, providing subsidies, and sensitizing the authorities directly related to the problem, aiming to solve it.	The sample was 449 children from 7 to 14 years born and always residing in Icém. The indices of Dean and Arnold, DMF and dmf were used. The physical-chemical tests revealed that one of the wells contained 4 mg F/l and the other 2.6 mg F/l, where it should contain 0.7 mg F/l.	Only 11.8% of the children were free of fluorosis, with few questionable (3.5%) and severe (0.06%) cases. There is a predominance of very mild degree (45.6%) and balance between mild and moderate degrees (18.9% and 19.3%, respectively). Children aged 9 to 11 years have the highest prevalence of dental fluorosis. The mean DMF was 2.60, classified as low.
Diniz and Cardoso (1979) [49]	Effectiveness study/Water	Compare the results of fluoridation of public water supplies between the cities of Juazeiro (BA) and Petrolina (PE), using calcium fluoride (fluorite).	A total of 1350 schoolchildren from 6 to 14 years old from Juazeiro and 900 from Petrolina were examined. Children born and resident since the time fluoridation started (1970) were included. The cost of fluoridation was calculated.	The percentage of caries-free schoolchildren in Juazeiro is higher than that of Petrolina (whose fluoridation was interrupted), and had an average reduction of 34.73%. The per-capita cost for Juazeiro was very low for the great benefit it provides.
Guimarães et al. (1980) [50]	Effectiveness study/Water, topical application of APF	Evaluate the reduction in caries incidence by the association of two preventive methods (annual topical application of acidulated sodium fluoride associated with fluoridation of public water supplies).	The sample of 177 schoolchildren aged 7 and 8 years, of both sexes, living and studying in areas with fluoridated water, and residing in Piracicaba since birth, was divided into 2 groups: control (did not receive topical fluoride application) and experimental (received an annual application for 2 consecutive years). The DMFT index was collected at the beginning and at the end of the study.	The annual topical application of acidulated sodium fluoride for 4 min, preceded by the cleaning, isolation and drying of teeth, associated with the fluoridation of public water supplies, is efficient in reducing caries incidence. In permanent teeth, there was a 22.7% reduction. The best orientation is to take preventive topical application programs to areas where there is no water fluoridation.
Cury et al. (1981) [51]	Monitoring/Toothpaste	Determine the concentration and analyze the forms of fluoride found in toothpastes sold in Brazil to assess the potential for preventing dental caries.	The fluoride content in the toothpastes Anticárie Xavier, Signal F, Kolynos SMF, Kolynos Gel F, Fluorgard and Pruf, purchased in the local market in Piracicaba, was potentiometrically analyzed. The manufacturing date is not indicated. The forms of fluoride were identified: free ionic fluoride (active in caries prevention), phosphate-bound fluoride MFP (active in caries prevention) and insoluble fluoride (bound to the abrasive and inactive in caries prevention).	The MFP concentration ranged from 657 to 902.3 ppm. The percentage of total fluoride ranged from 65 to 96%. Most of the dentifrices have a total fluoride concentration close to that indicated by the manufacturer. All the analyzed toothpastes commercialized in Brazil have a MFP concentration sufficient to prevent caries. However, as MFP breaks down over time, it is necessary to prove the stability of the dentifrice so that the prevention potential is preserved.
Moitta, F. (1981) [52]	Review/Water	Show the status of water fluoridation in Brazil from the beginning in 1953 to the present.	More than 30 cities (20 million people) have benefited. There is mention of the fluoride compounds used and the contribution of FSESP, the Ministry of Health and the National Institute of Food and Nutrition (INAN). The research of new fluoridation methods and the national production of fluorosilicic acid were encouraged.	A little more than 10% of Brazil’s population benefits from the measure. To reduce caries, the measure must be intensified and extended to populations in need. The Federal and State Governments should become aware of the socioeconomic importance of the measure and promote it, ensuring support and resources.
Araújo, IC. (1982) [53]	Fluoride use in school programs/Mouthwashes, topical application of sodium fluoride	Plan and implement the dental health policy through incremental treatment, reaching, in a staggered and compulsory way, schoolchildren from 6 to 14 years old.	The focus was children from 6 to 14 years of age, from the schools where the project will be implemented gradually. The maintenance service will be the responsibility of the neighborhood Health Unit.	The activities planned by the Health Secretary are guided by norms and instructions, taking into account the following aspects: hierarchization of the dental problem; establishment of priority criteria; standardization of equipment and supplies; service supervision; statistical data collection; production and productivity evaluation, and development of oral health educational actions.
Diniz et al. (1982) [54]	Economic aspects/Water	Research a simple and economical process of fluoridation of water supplies for small communities through the use of fluorite.	Technical and scientific criteria of a simplified water fluoridation technique developed for small communities are presented. Calcium fluoride (fluorite) was used as basic material, following the natural content of fluoride in water.	This technique assumes low cost, ease of operation, and use of locally available natural resources.
Vasconcellos, M. C. C. (1982) [55]	Effectiveness study/Water	Verify the caries prevalence and the level of dental care with respect to the disease in the urban school population of Araraquara.	The sample was 9923 schoolchildren from 7 to 12 years. The examinations were conducted in November 1979 and the DMFT index was used.	The DMFT index values are higher than those expected for a community whose public water supply has been fluoridated for approximately 16 years. The data strengthen the hypothesis of discontinuity in the concentration of fluoride in water.
Pinto, V. G. (1982) [56]	Review/Salt	To discuss the feasibility of using table salt as a carrier for fluoride in caries prevention in Brazil.	It considers several points such as the Colombian study, the Brazilian position favoring water fluoridation, salt iodation and individual variations in salt intake.	In view of the current scientific knowledge and the peculiarities of Brazil, the widespread adoption of fluoridated salt for caries prevention is not justified. The use of fluoridated salt is valid only in restricted areas and under technical control. Water fluoridation is the method of choice for the country, which should strive for its maximum expansion.
Buendia, O.C. (1983) [57]	Fluoride use in school programs/Water	1. Verify the ideal fluoride content for maximum benefit in reducing caries in schoolchildren. 2. Verify the possibility of appearance of dental fluorosis, due to the ingestion of higher doses of fluoride. 3. Evaluate the strength of the method in reducing caries incidence.	Four studies were analyzed, conducted with fluoridation of school water in four cities with different annual average temperatures and varying the fluoride concentration applied. The results after 6, 8 and 12 years are presented.	The recommended content to fluoridate school water is 4.5 times higher than that indicated in municipal water. The reduction in caries incidence in children ranges from 34.9 to 39.7% with 8 years of implementation. Water fluoridation in schools is a safe and effective method.
Pinto, V.G. (1983) [58]	Review/Water, mouthwash, topical application	Understand the current situation in the country in terms of the dental situation as a whole, by gathering data and indicators, directly or indirectly related to dental health, and critically analyzing them.	It provides a Brazilian dental health panorama, with emphasis on the economic and epidemiological situation, caries prevention, human resources, financial expenditures and services delivery structure.	It suggests guidelines to manage the Dentistry Program. Foresees the changes and criteria that a new dental policy for Brazil should include throughout this decade. It is expected that, by the year 2000, about 85% of the urban population have treated water and half with fluoride.
Buendia, O.C. (1984) [59]	Effectiveness study/Mouthwash	Comment on the advantages of using fluoride mouthwashes in the prevention of dental caries.	A sodium fluoride solution was applied with a weekly technique employed in 494 municipalities in SP, having benefited 1,665,364 students until December 1982.	Fluoride mouthwashes use is an alternative solution for people living in places without fluoridated water, for being a simple and easy method to be applied. With 2 years of usage, the average caries reduction was 35%.
Viegas and Viegas (1985) [60]	Effectiveness study/Water	Verify the reductions in dental caries in primary and permanent teeth during 10 years of fluoridation of public water supplies.	Children from 3 to 14 years old and young adults from 15 to 19 years old who have always lived in the city of Barretos, SP, were examined. Evaluating decayed, filled, lost, healthy teeth, caries in the lower right permanent first molar and upper central incisors.	There was a reduction in the mean DMF at all ages. In the group of 3 to 5 years, 52% are caries free. In the group of 6 to 14 years, the percentage of healthy and restored teeth increased, and the percentage of decayed and extracted teeth decreased. In the 6 to 10 age group, there was a 55% reduction in caries. In the 15 to 19 age group, there was a 100% reduction in the need for dentures.
Lacerda, J. L. S. (1985) [61]	Effectiveness study/Water	Ratify the importance of fluoride therapy for caries prevention through water fluoridation with fluorite in Minas Gerais.	The data collected by the epidemiological dental caries surveys were compared using the DMF index before and after fluoride.	In some cities, there was a caries reduction of between 64 and 73%. There was a decrease in the average number of decayed permanent teeth and an increase in restored permanent teeth. There was a high percentage of children with zero caries (33 to 39%).
Bastos et al. (1985) [62]	Adverse effects of fluoride/Supplement	Analyze fluoride intake during the calcification phase of teeth, discussing fluorosis and prescribing the amount to be administered daily for pregnant women and children.	The recommended dosage for pregnant women and children according to some authors and according to the ADA is 0.50 mg F/day. Fluorosis will only develop with the average ingestion of 2 ppm or more, and the risk only exists if it is consumed systemically.	A dosage of 0.50 mg F/day is recommended for children up to 3 years old, and 1 mg F/day for children older than 3 years. For pregnant women, the recommended dosage is 1 mg F/day. Given the very high caries prevalence in Brazil, fluoride supplements can and should be prescribed by a doctor or dentist in regions without fluoridated water.
Viegas and Viegas (1985) [63]	Effectiveness study/Water	Present and analyze the prevalence data of dental caries verified in the study of water fluoridation in Campinas.	The prevalence data of caries in schoolchildren aged 4 to 14 years were analyzed to verify the reductions in caries during 14 years of water fluoridation. The methodology and technique were the same used in the 4 previous surveys (1965, 1969, 1972 and 1976).	The reductions in caries prevalence were 57% in permanent teeth and 49% in primary teeth. In children aged 4 to 14 years, 26% have no decayed teeth, and in children aged 5 to 14, 29% are in the same condition as for permanent teeth.
Martins et al. (1985) [64]	Effectiveness study/Water	Analyze clinically, radiographically and statistically the caries prevalence in deciduous teeth in Belo Horizonte MG, whose public water supply has the ideal fluoride concentration, and in Rio Acima, MG, whose water does not contain fluoride.	The sample consisted of 742 schoolchildren aged 7 to 10 years, of both sexes, of low socioeconomic level, without distinction of color. There were 450 from Belo Horizonte (0.76 ppm F) and 292 from Rio Acima (0.10 ppm F). Water samples were collected in both cities. The dmfs index was used.	The caries prevalence in deciduous teeth is higher in Rio Acima children than in Belo Horizonte children. There was a 33.18% caries reduction in deciduous teeth in children who always used water with the ideal fluoride concentration.
Bastos, J.R.M. (19859 [65]	Effectiveness study/Supplement	Show the use of fluoride supplementation as an alternative method of caries prevention.	In a country with the highest caries rate in the world, it is important to indicate fluoride for pregnant women and children, trying to reduce caries in the population as much as possible.	In a non-fluoridated area or with low F concentration (1.5 to 2 mg F/day), supplements should be prescribed. In areas with unsatisfactory fluoride levels, the supplement can potentiate the effect of fluoride.
De Pretto et al. (1985) [66]	Effectiveness study/Water	Present the results after 8 years of fluoridation and describe current techniques for fluoride use in semi-artesian wells in Bauru, SP.	The processes and costs of fluoridation in the benefited regions are explained. A dental caries survey was conducted in 1984 on 2416 students from 8 elementary schools located in area with fluoridated water, after 8 years of water fluoridation. Another survey was carried out in 1976 (1515 schoolchildren). The DMFT index was used only for permanent teeth.	The number of teeth attacked by caries was lower in 1984. The reduction ranged from 29 to 36% according to age. The possible causes of this difference are children living in areas without fluoridated water, the habit of families to use water from artesian wells even with the availability of fluoridated water and interruptions of fluoridation until 1981. It is recommended to extend the fluoridation system to the entire city and to extend an educational campaign for parents to understand the importance of water quality and fluoridation. It is safe and no harmful effects have been noted at 0.9 ppm.
Cury, J.A. (1986) [67]	Monitoring/Toothpaste	Study the stability of fluoride in dentifrices sold in Brazil in terms of caries prevention potential.	Seven toothpastes were analyzed at the time of purchase and after 6 and 12 months of storage in their original packaging and at room temperature. The fluoride content and its different chemical forms (free ionic fluoride, MFP and insoluble fluoride) were determined.	In fluoride-containing toothpastes, its concentration decreased, increasing the percentage of fluoride bound to the abrasive. In MFP-based toothpastes, it remained stable or underwent hydrolysis, increasing the concentrations of fluoride bound to the abrasive. In some toothpastes, this instability may compromise their cariostatic effect.
Teixeira, R.N and Cury, J.A. (1986) [68]	Monitoring/Toothpaste	Test the reactivity of the fluoride present in toothpastes sold in Brazil, providing a comparative and complementary analysis.	Toothpastes purchased in the local commerce of Piracicaba, SP, were evaluated in vitro. The concentrations of total fluoride, calcium fluoride and fluoridated apatite were determined in the enamel after reactions with the toothpaste solutions.	Toothpastes have been shown to be highly efficient in their ability to form calcium fluoride in the enamel. Given the importance of toothpastes in the decline in caries prevalence in industrialized countries, it is up to dentistry to demand quality control from the factories and the government, so that the same can occur in Brazil.
Vertuan, V. (1986) [69]	Effectiveness study/Water	Compare the mean caries index before fluoridation and 19 years after fluoridation started.	Sample of 639 schoolchildren aged 7 to 12 years from 9 schools. All drank fluoridated water since birth and always lived in Araraquara SP. The DMF index was used.	There was a 41.8% reduction in caries incidence after 19 years of water fluoridation, although a better result was expected. Compared to the data from 1972, there was an increase in the caries rate in the group studied. There is a need for greater concern and priority to maintain optimal standards of fluoride in water to achieve better oral health.
Zamorano et al. (1987) [70]	Effectiveness study/Water	Compare the prevalence of caries in permanent teeth in Belo Horizonte (0.76 ppm F) and Rio Acima (0.10 ppm F).	Sample of 946 schoolchildren (567 from Belo Horizonte and 379 from Rio Acima) of both sexes, between 7 and 11 years, of low socioeconomic level, without distinction of race, who were born, had always resided and consumed water from public supplies in the two cities. Clinical and radiographic exams were performed.	Caries had an average reduction of 51.64% in both cities. The difference between the means of the DMFS index is lower in Belo Horizonte. Caries is a progressive and cumulative disease.
Viegas et al. (1987) [71]	Advocacy/Water	It is a document in response to an narrative that TV Globo presented about the fluoridation of public supply water in March 29, 1987.	There were 3 questions asked in the program: Is fluoride toxic? Is fluoride good, but you do not know what will happen 20 years from now? Is fluoridation of the public water supply an approved method? Or is it still necessary to debate the issue to reach a conclusion?	There is no scientific basis for the criticisms that were made about water fluoridation in the TV show. It is necessary to stress that this method has been exhaustively studied and is approved by the international scientific community and should be an integral part of the National Health Policy, as a condition to have better Dental Health for the population of Brazil.
Nobre dos Santos and Cury (1988) [72]	Effects of cessation/Water	Report the alteration of fluoride concentration in plaque after discontinuation of water fluoridation in Piracicaba, SP, Brazil.	The water was fluoridated in 1971. Fluoridation was discontinued in 1987 due to a shortage of sodium fluorosilicate. Dental plaque was collected from 91 children of both sexes, 6 to 8 years of age, during the last 6 months of water fluoridation (0.8 ppm F) and from 41 children after its cessation (0.06 ppm F).	The fluoride concentration in the plaque 2 months after stopping water fluoridation was lower than during fluoridation. Discontinuation of water fluoridation may contribute to the reduction in the cariostatic effect due to the interruption in fluoride intake.
Cury, J.A. (1988) [73]	Monitoring/Toothpaste	Evaluate the fluoride of two dentifrices that offer chemical control of dental plaque.	Toothpastes of the brands Colgate Antiplaca and Prevent were evaluated. A non-fluoride dentifrice was used as a control. The reactivity of fluoride in the toothpastes with human tooth enamel was verified by assessing the total fluoride ion concentrations (TF), in the form of calcium fluoride and in the enamel after the reaction with the toothpastes simulating brushing.	In the event of failure of the dentifrices in the mechanochemical control of dental plaque, the efficiency in caries prevention will be better ensured by the Prevent dentifrice, which has fully available, stable and more reactive fluoride than Colgate Antiplaque.
Viegas and Viegas (1988) [74]	Effectiveness study/Water	Verify dental caries reductions found during the sixteen-year period of public water supply fluoridation.	The results of the prevalence data of dental caries in children aged 5 to 14 years and adults aged 15 to 24 years from the city of Barretos, SP, Brazil, were analyzed.	In the 5- and 6-year-old children, 66.1% had no decayed teeth; in the 6- to 14-year-olds, there was a 54% reduction in the average DMF; and in the 12-year-olds, the average DMF was 3.5. In the 18-year-olds, 72.3% had all their teeth, and in the 15- to 24-year-olds, there was a 90.25% reduction in the need for dentures.
Lopes et al. (1988) [75]	Effectiveness study/Water	Compare the prevalence of caries in permanent first molars of children who were born and have always resided in two communities in the state of Piaui (one with fluoridated water and the other without).	The DMFS index of the first permanent molars was used. These teeth were examined clinically and radiographically in a sample of 360 schoolchildren aged 7 to 12 years, of both sexes, of low socioeconomic status, without distinction of race, who were born and had always resided in the cities of Teresina and Barras, PI, the first, with fluoride in the public water supply (0.68 ppm F) and the second without fluoride.	For all ages and sexes, in children in the city without fluoride, prevalence rates were always higher. The percentage reduction at all ages, in both sexes and in both communities was between 19.38% and 38.09%. This may be explained by an under-fluoridation reported in 1985–1986 in the public water supply of Teresina (0.58 ppm and 0.57 ppm). The DMFS index of Barras was higher when compared to Teresina.
Arcieri et al. (1988) [76]	Effectiveness study/Gel, mouthwash	Evaluate the reduction in the incidence of caries through the association of two preventive methods.	A total of 246 students of both sexes, aged 7 to 11 years, from Uberlândia, were examined. They were divided into 2 groups: Group I received half-yearly topical applications of 1.23% APF; and Group II, in addition to the half-yearly topical applications, received weekly mouthwashes with 0.2% sodium fluoride aqueous solution.	After 2 years, group II had a 33.97% reduction in the incidence of caries in permanent teeth. Weekly mouthwashes associated with two annual topical applications of fluoride are effective in reducing the incidence of caries in schoolchildren aged 7 to 11 years.
Neder and Manfredini (1991) [77]	Review/Salt	Discuss the sudden launch of the National Program for Caries Control by the Salt Fluoridation Method in Brazil.	The authors considered the Health Minister’s announcement to be irresponsible, 30 years after the implementation of water fluoridation. It would imply importing potassium fluoride from a single producer in Germany and equipment for homogenization; solving operational problems such as correct addition and adequate quality control; differentiated distribution respecting the areas with natural or artificial fluoride in the water; making necessary previous studies such as the food survey evaluating salt consumption in the country by region and social class, the cost not as low as expected and the organization of sanitary control in distribution.	Any decision to change the method of fluoridation used in the country should be subject to prior discussion between public institutions and the citizens involved, as well as the National Congress, which is responsible for making decisions on the use of fluoride as a caries prevention measure in the country.
Silva, M.F.A. (1991) [78]	Review/Salt	Analyze the conditions of fluoride use in salt, discussing whether there are conditions for using this method in Brazil.	Its effectiveness is similar to that of water fluoridation and has a very low cost; however, it has technical and dosage control problems. There are problems with the dosage of iodine in salt, observing variations of 30%. If this happened with fluoride, the effect of caries prevention would not be observed or could cause fluorosis. This could make the program unviable and could even cause harm to the supervision of programs such as water fluoridation. There are other problems such as the analysis of the natural fluoride content in salt, the interference of impurities in the salt that may react with fluoride and the control of humidity and flow.	Fluoridated salt is a cheap and effective method for caries control. However, only 27% of the salt produced in Brazil is refined, so a large part of the population consumes unrefined salt. Because of the large number of companies producing salt, it is difficult to control the product at a national level. Companies are not interested in responsible associations with health programs. The observed variation in iodine concentration in salt apparently has no side effects, which would not occur with fluoride. There is a lack of studies on salt intake per capita by region regarding the conditions under which salt is supplied to populations (humidity, packaging, etc.). It would be imprudent to replace a method widely accepted by the population and widely tested nationally and internationally, for one that needs studies due to the peculiar conditions of distribution and consumption of salt in Brazil.

**Table 6:** Normative documents related to the use of fluorides for dental caries prevention in Brazil in the pre-constitutional reform period.

Title	Date	Institution	Vehicle	Topic	Orientation/Recommendation/Declaration	
Ordinance 33 of 28 May 1968 [79]	1968	National Health Department/Ministry of Health/National System of Dentistry Fiscalization	Water	Promotes a study to formulate an Executive Plan for Fluoridation of Supply Water.	Set up study committees composed of three dental surgeons to be appointed by this service.	
Law 6050 of 24 May 1974 [80]	1974	National Congress	Water	Disposes about the fluoridation of water in supply systems when there is a treatment plant.	Projects for the construction or expansion of public water supply systems, where there is a treatment plant, must include forecasts and plans for water fluoridation.	
Decree 76,872 of 22 December 1975 [81]	1975	Presidency of the Republic, Civil House, Deputy Chief of Staff for Legal Affairs	Water	Regulates Law 6050 of 1974, which provides for the fluoridation of water in public supply systems.	Projects for the construction or expansion of public water supply systems must contain studies on the need for fluoridation of water for human consumption. Even in systems that do not have a treatment plant, where appropriate fluoridation methods and processes must be used.	
Ordinance 635 of 26 December 1975 [82]	1975	State Minister of Health	Water	Approves norms and standards on water fluoridation, in view of Law 6050 of 1974.	It details the minimum requirements: continuous water supply, the potability standards, adequate operation and maintenance systems, and routine control of water quality.	
Decree 90,892 of 1 February 1985 [83]	1985	Presidency of the Republic. Government of the Argentine Republic, the United Mexican States and the Federative Republic of Brazil	Gel	Provides for the execution of trade agreement No. 26 (Brazil’s agreement with Argentina and Mexico), signed in the industry sector of hospital, medical, dental, veterinary and related articles and devices.	On imports of the specified products originating in Argentina, Mexico and Bolivia, Ecuador and Paraguay. Chapter I of the agreement is on industrial sector products, including sodium fluoride gel (caries preventive).	
Law 7486 of 6 June 1986 [84]	1986	Presidency of the Republic.	Water	Approves the guidelines of the first National Development Plan (PND) of the New Republic, for the period from 1986 to 1989.	Enhancement of the mass prevention program of fluoride in public water supplies.	
Ordinance 21 of 25 October 1989 [85]	1989	Ministry of Health/National Health Surveillance System	Mouthwashes and toothpastes	On the registration of fluoride mouthwashes and toothpastes and the fluoride compounds accepted in the formulation of these products.	Mouthwashes: F concentration (202.5 to 247.5 ppm), fluoride compounds and label. Toothpastes: F concentration (1000 to 1500 ppm), fluoride compounds and label.	
Ordinance 22 of 20 December 1989 [86]	1989	Ministry of Health/National Health Surveillance System	Mouthwashes and toothpastes	On the registration of fluoride mouthwashes and toothpastes and the fluoride compounds accepted in the formulation of these products.	Mouthwashes: F concentration (202.5 to 247.5 ppm), fluoride compounds and label. Toothpastes: F concentration (1000 to 1500 ppm), fluoride compounds and label.	
Ordinance 1437 of 14 December 1990 [87]	1990	Ministry of Health/Minister’s Cabinet	Salt	Creates the National Program for Caries Control by the salt fluoridation method.	It proposes implantation of the Program in the North, Northeast, and Center–West regions.	
Ordinance 101 of 31 January 1991 [88]	1991	Ministry of Health/Minister’s Cabinet	Salt	Alters clauses II and III of article 2 and clauses II of article 3 of ordinance 1437 of 1990.	It establishes the Program Execution Coordination, as well as the Evaluation Coordination and the General Coordination’s function of elaborating the technical norms for the program’s development.	
Ordinance 1 of 5 February 1991 [89]	1991	INAN/ Ministry of Health	Salt	Determines attributions for the General Coordinator of the National Program for Caries Control by the salt fluoridation method.	Take responsibility for the program, designate its organizational structure, organize the program’s annual budget proposal and nominate the Technical Advisory Group to the Ministry of Health.	
Ordinance 2 of 28 February 1991 [90]	1991	INAN/Ministry of Health	Salt	The technical norms for the development of the National Program of Caries Control by the salt fluoridation method, are approved.	Technical Standards: Presentation, Execution, Operational mechanisms of execution, Operational and monitoring mechanisms, Program evaluation mechanism.	
Ordinance 3 of 13 August 1991 [91]	1991	INAN/Ministry of Health	Salt	Changes item 7, from clause III, of the technical norms attached to the Ordinance 2, 1991, from the General-Coordination of the National Program for Caries Control by the salt fluoridation method.	Increases the deadline for all refineries in the North, Northeast and Center–West regions to comply with what Ordinance 1437 of 1990 establishes.	
Ordinance 851 of 4 August 1992 [92]	1992	Ministry of Health/Minister’s Cabinet	Salt	Cancellation of the National Program for Caries Control by the Salt Fluoridation Method.	Makes unsubstantiated the Ordinance 1437 of 1990 and other acts.	
